# Antibody evasiveness of SARS-CoV-2 subvariants KP.3.1.1 and XEC

**DOI:** 10.1016/j.celrep.2025.115543

**Published:** 2025-04-08

**Authors:** Qian Wang, Yicheng Guo, Ian A. Mellis, Madeline Wu, Hiroshi Mohri, Carmen Gherasim, Riccardo Valdez, Lawrence J. Purpura, Michael T. Yin, Aubree Gordon, David D. Ho

**Affiliations:** 1 Aaron Diamond AIDS Research Center, Columbia University Vagelos College of Physicians and Surgeons, New York, NY, USA; 2 Pandemic Research Alliance Unit at the Wu Center for Pandemic Research, Columbia University Vagelos College of Physicians and Surgeons, New York, NY 10032, USA; 3 Department of Pathology and Cell Biology, Columbia University Vagelos College of Physicians and Surgeons, New York, NY 10032, USA; 4 Department of Pathology, University of Michigan, Ann Arbor, MI 48109, USA; 5 Division of Infectious Diseases, Department of Medicine, Columbia University Vagelos College of Physicians and Surgeons, New York, NY, USA; 6 Department of Epidemiology, University of Michigan, Ann Arbor, MI 48109, USA; 7 Department of Microbiology and Immunology, Columbia University Vagelos College of Physicians and Surgeons, New York, NY 10032, USA

**Keywords:** SARS-CoV-2, JN.1 subvariants, KP.3.1.1, XEC, mRNA vaccines, serum neutralization, monoclonal antibodies, antibody evasion, ACE2 inhibition

## Abstract

Severe acute respiratory syndrome coronavirus 2 (SARS-CoV-2) continues to evolve and spread, and it remains critical to understand the functional consequences of mutations in dominant viral variants. The recombinant JN.1 subvariant XEC recently replaced KP.3.1.1 to become the most prevalent subvariant worldwide. Here, we measure the *in vitro* neutralization of KP.3.1.1 and XEC by human sera, monoclonal antibodies, and the soluble human ACE2 (hACE2) receptor relative to the parental subvariants KP.3 and JN.1. KP.3.1.1 and XEC are slightly more resistant (1.3- to 1.6-fold) than KP.3 to serum neutralization and antigenically similar. Both also demonstrate greater resistance to neutralization by select monoclonal antibodies and soluble hACE2, all of which target the top of the viral spike. Our findings suggest that the upward motion of the receptor-binding domain in the spike may be partially hindered by the N-terminal domain mutations in KP.3.1.1 and XEC, allowing these subvariants to better evade serum antibodies that target the viral spike in the up position and to have a growth advantage.

## Highlights

• KP.3.1.1 and XEC are 1.3- to 1.6-fold more resistant to serum neutralization than KP.3
• KP.2 MV booster may elicit higher titers against KP.3.1.1 and XEC than JN.1 infection
• Spike mutations F59S and S31Δ are functionally similar for receptor binding and mAb evasion

## Graphical abstract

**Figure:**
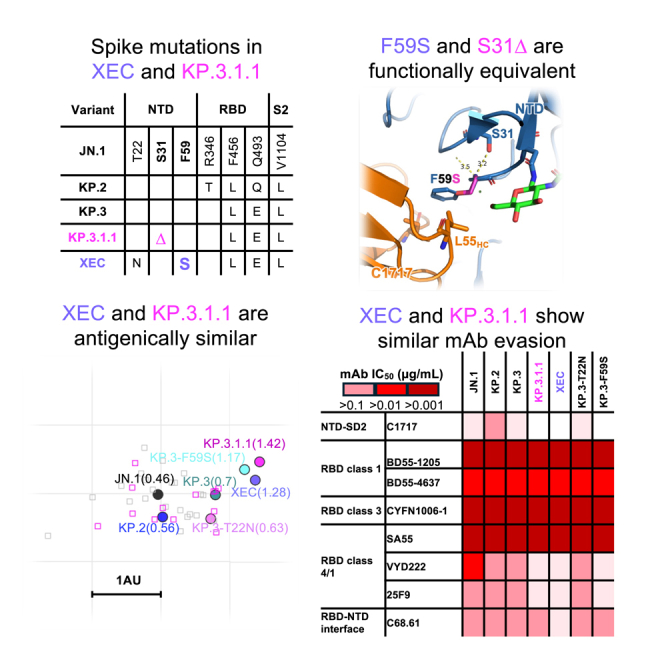

## Teaser

SARS-CoV-2 Omicron JN.1 subvariants KP.3.1.1 and XEC emerged as dominant over parental strains. Wang et al. show that KP.3.1.1 and XEC are similarly antibody evasive due to different spike NTD mutations at interacting amino acids, which likely leads to similar impairments of spike conformational changes.

## Introduction

**Figure 1:**
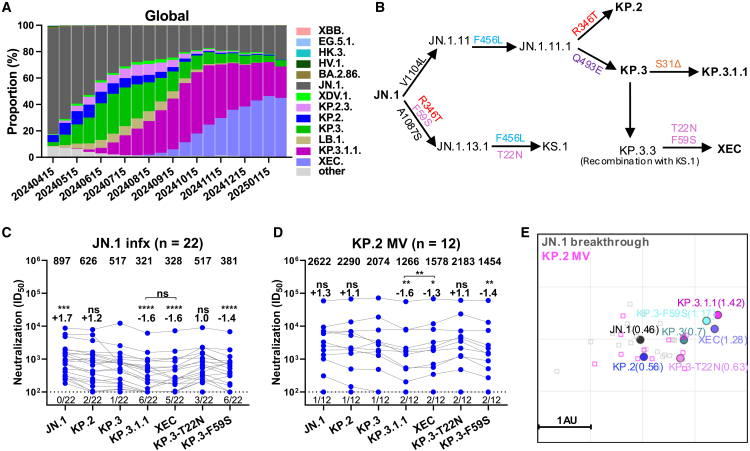
Characterization of SARS-CoV-2 JN.1 sublineages, including serum neutralizing antibody evasion and antigenicity (A) Relative frequencies of dominant SARS-CoV-2 subvariants from April 2024 to February 2025; data are from GISAID.^1^ (B) Viral evolutionary pathways and spike mutations of the indicated JN.1 subvariants. (C and D) Serum neutralizing titers (ID_50_) against VSV-based pseudoviruses bearing spike proteins from SARS-CoV-2 JN.1 sublineages for samples from cohorts JN.1 infx (C) and KP.2 MV (D). Compared with KP.3, KP.3.1.1 carries an S31Δ spike mutation in the NTD. The geometric mean ID_50_ titer (GMT) is presented at the top. The fold change in GMT for each virus compared to KP.3 is also shown immediately above the symbols. Statistical analyses used Wilcoxon matched-pairs signed-rank tests, comparing to KP.3. *n*, sample size; ns, not significant. ^∗^*p* < 0.05, ^∗∗^*p* < 0.01, ^∗∗∗^*p* < 0.001, ^∗∗∗∗^*p* < 0.0001. Numbers under the dotted lines denote numbers of serum samples that were under the limit of detection (ID_50_ < 100). (E) Antigenic map generated using all neutralization data from (C) and (D). One antigenic unit (AU) represents an approximately 2-fold change in ID_50_ titer. Serum samples and viruses are shown as squares and dots, respectively. The geometric mean antigenic distance of variants to sera is indicated in brackets.

The severe acute respiratory syndrome coronavirus 2 (SARS-CoV-2) Omicron JN.1 subvariant rapidly increased in prevalence around the world starting in late 2023, and its progeny sublineages KP.2 and KP.3 were dominant successively and briefly thereafter. Recently, KP.3.1.1, bearing an S31 deletion (S31Δ) on top of the KP.3 spike, was dominant worldwide through October 2024^1^ (Figure 1A). Since then, SARS-CoV-2 infections due to XEC, a recombinant of JN.1 subvariants KS.1 and KP.3.3, have become most frequent. XEC carries two additional spike N-terminal domain (NTD) mutations T22N and F59S beyond those in KP.3 (Figure 1B; Table S1). How these spike mutations confer a growth advantage to KP.3.1.1 and XEC remains unknown.

## Results

### Serum neutralizing antibody evasion and antigenicity of KP.3.1.1 and XEC

To address this question, we first tested serum neutralization against vesicular stomatitis virus (VSV) pseudotyped KP.3.1.1, XEC, and XEC individual spike mutations on KP.3 (KP.3-T22N and KP.3-F59S) compared to JN.1, KP.2, and KP.3, using samples from two cohorts of adults: (1) participants with a history of JN.1 sublineage infection during 2024, sampled 32–87 days after infection (“JN.1 infx”), and (2) participants who received an updated KP.2-based mRNA monovalent vaccine booster, sampled approximately 4 weeks after dosing (“KP.2 MV”) (Tables S2 and S3). The majority of participants were female. Compared with KP.3.1.1, XEC demonstrated a similar level of evasion to serum neutralization in the JN.1 infx cohort (Figure 1C) but was slightly more sensitive to serum neutralization in the KP.2 MV cohort (Figure 1D). KP.3 was 1.3- to 1.7-fold more resistant to serum neutralization than JN.1, while KP.3.1.1 and XEC were 1.3- to 1.6-fold more resistant than KP.3. Critically, we found that these increases in resistance to serum neutralization were explained by the component mutations S31Δ and F59S, individually tested on the background of KP.3 as KP.3.1.1 and KP.3-F59S, respectively (Figures 1C and 1D). In addition, serum neutralizing titers in KP.2 MV participants were generally higher than in JN.1 infx participants, with levels correlated with clinical protection.^2^

To compare the antigenicity of the tested subvariants, the serum neutralization data from both cohorts were used to generate antigenic maps. We observed that KP.3.1.1, XEC, and KP.3-F59S clustered closely together, approximately 1.2 antigenic units from JN.1, while KP.3 and KP.3-T22N exhibited a shorter but similar antigenic distance to JN.1 (Figure 1E). These results indicate that F59S and S31Δ are comparable antigenically, while T22N has minimal impact on neutralization by these sera, consistent with other reports on the antigenicity of XEC and KP.3.1.1.^3^^,^^4^

### Monoclonal antibody evasion and receptor binding of KP.3.1.1 and XEC

**Figure 2:**
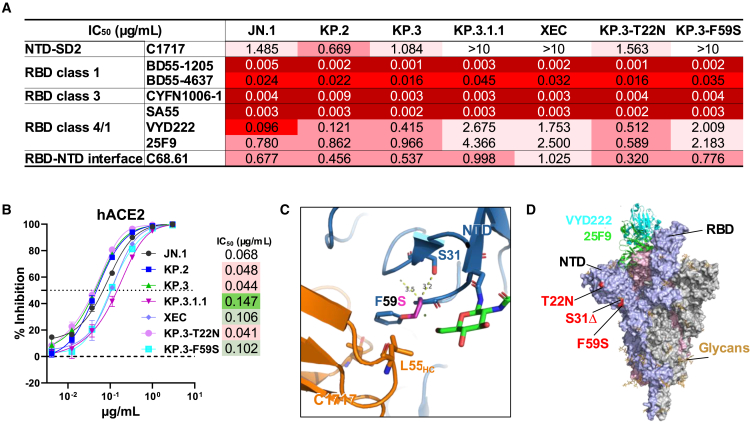
mAb neutralization, hACE2 inhibition, and structural analysis of the indicated JN.1 sublineage variants (A) mAb neutralization against the indicated pseudoviruses. Antibody concentrations resulting in 50% inhibition of infectivity (IC_50_) are presented. (B) Sensitivity of JN.1 subvariants to hACE2 inhibition. IC_50_ values are also presented. Data are shown as mean ± SEM for three technical replicates. (C) Structural analysis of S31Δ and F59S in the complex of C1717 and NTD (PDB: 7UAR). (D) Structural analysis of T22N, S31Δ, and F59S in the SARS-CoV-2 spike (PDB: 7KRR), shown in complex with VYD222 (cyan; PDB: 7U2D) and 25F9 (green; PDB: 8GB5).

Next, to evaluate which epitopes were most closely associated with the antibody evasion of KP.3.1.1 and XEC, we performed neutralization assays using a panel of monoclonal antibodies (mAbs) that retained potency against KP.3, directed to multiple epitopes on the viral spike. S31Δ and F59S knocked out the NTD-SD2-specific antibody C1717^5^ and impaired the receptor-binding domain (RBD) class 4/1 antibodies VYD222 (pemivibart)^6^ and 25F9,^7^ potentially explaining the increased resistance of KP.3.1.1 and XEC to serum neutralization (Figure 2A), consistent with other reports using distinct panels of mAbs.^3^ But how do mutations at the bottom of the NTD affect mAbs directed to the top of the spike? To address this question, we tested the inhibition of soluble human ACE2 (hACE2) against the same panel of pseudoviruses. T22N did not alter the susceptibility of KP.3 to hACE2 inhibition, while S31Δ and F59S impaired hACE2 inhibition by 3.3- and 2.3-fold (Figure 2B), respectively, indicating a lower affinity for the viral receptor. These findings collectively suggested that the upward motion of the RBD may be impaired by either S31Δ or F59S, since the spike binding to both the receptor and class 4/1 antibodies requires the RBD to be in the up position. Indeed, structural analysis showed that S31 and F59 interact via hydrogen bonding in both the up and down conformations (Figure 2C) and that S31Δ and F59S mutations were functionally equivalent in altering NTD conformation, not only enabling escape from NTD-SD2-directed antibodies like C1717 but also likely indirectly hindering the upward movement of the RBD. S31Δ and F59S, distant from the RBD, therefore likely indirectly impair binding of class 4/1 antibodies, in addition to their more direct interference with NTD-directed antibodies (Figure 2D). The conformational alterations caused by S31Δ and F59S may further enhance the immune evasion trend driven by F455S, F456L, and Q493E in the evolution of JN.1 sublineages (Figure 1B), which have already led to a large proportion of weak class 4/1 antibodies in the repertoire. This pattern recapitulates previous observations in XBB lineages, where a greater proportion of the RBD-down conformation in later subvariants contributes to immune evasion by conformational masking of immunodominant RBD regions.^8^

## Discussion

In summary, KP.3.1.1 and XEC demonstrate greater antibody evasion than JN.1 and KP.3, which likely contributes to their increasing global prevalence. The functional equivalence of S31Δ and F59S mutations renders KP.3.1.1 and XEC antigenically similar. The results here are consistent with most other posted reports using different clinical cohorts,^4^^,^^9^^,^^10^^,^^11^^,^^12^ and they extend beyond posted or published data in several ways. Specifically, we show neutralizing titers in a cohort of KP.2 mRNA vaccine-boosted human participants, which have not been reported previously in the literature, to the best of our knowledge. Furthermore, our results based on neutralization with a large panel of mAbs, combined with hACE2 inhibition assays and structural modeling, provide evidence of up-conformation impairment as a common functional consequence of S31Δ and F59S mutations. Lastly, our results show that XEC is more resistant to neutralization by the US Food and Drug Administration-authorized VYD222 antibody, consistent with our prior results for KP.3.1.1,^13^ so the clinical efficacy of VYD222 should be monitored closely as XEC increases in prevalence.

### Limitations of the study

There are several factors that may limit the generalizability of our findings. First, there were relatively fewer participants in the KP.2 MV cohort compared to the JN.1 infx cohort, so we may not be able to detect smaller differences between these two groups. Second, we use human ACE2 pseudovirus inhibition to assess receptor binding, but we do not have results from orthogonal methods for accessing receptor binding. Third, we infer conformational change impairments due to F59S and S31Δ due to mAb neutralization results, receptor binding assay results, and computational structural analyses, but we do not present direct biochemical evidence of differences in the frequency of up vs. down spike conformational changes.

## Resource availability

### Lead contact

Requests for further information, resources, and reagents should be directed to and will be fulfilled by the lead contact, David D. Ho (dh2994@cumc.columbia.edu).

### Materials availability

All reagents generated in this study are available from the lead contact with a completed materials transfer agreement.

### Data and code availability

• Data reported in this paper will be shared by the lead contact upon request.
• This paper does not report original code.
• Any additional information required to reanalyze the data reported in this paper is available from the lead contact upon request.

## Acknowledgments

This study was supported by funding from the NIH SARS-CoV-2 Assessment of Viral Evolution (SAVE) Program (subcontract 0258-A700-4609 under federal contract 75N93021C00014 to D.D.H. and subcontract GR0010139-PO024016 under federal contract 75N93021C00016 to A.G.) and the Gates Foundation (project INV019355 to D.D.H.), internal startup funding (UR014016) from 10.13039/100006474Columbia University (to Y.G.), K23 AI171263 (to L.J.P.), and K24 AI155230 (to M.T.Y.). We thank all who contributed their data to the Global Initiative on Sharing All Influenza Data (GISAID).^1^ We express our gratitude to Jayesh Shah, Amanda Castillo, Meredith McNairy, and Antonia Sturizo for conducting the C-PIC study (Columbia) and Zijin Chu, Theresa Kowalski-Dobson, Anna Buswinka, Gabe Simjanovski, Joseph Wendzinski, Mayurika Patel, Kathleen Lindsey, and Dawson Davis of the VIVA study team for conducting the VIVA study.

## Author contributions

The study was conceptualized by Q.W., Y.G., I.A.M., and D.D.H. Experiments were conducted and data analyzed by Q.W., Y.G., I.A.M., and M.W. Project management was handled by Q.W. Serum samples were collected by H.M., C.G., R.V., L.J.P., M.T.Y., A.G., and their colleagues. The results were analyzed and the manuscript was written by Q.W., Y.G., M.W., I.A.M., and D.D.H. All contributing authors have reviewed and endorsed the manuscript.

## Declaration of interests

D.D.H. co-founded TaiMed Biologics and RenBio, and he serves as a consultant for WuXi Biologics and Brii Biosciences and is a board director at Vicarious Surgical. A.G. served as a member of the scientific advisory board for Janssen Pharmaceuticals and has consulted and serves on a scientific advisory board for Sanofi Pasteur.

## STAR★Methods

### Key resources table

**Table:** 

REAGENT or RESOURCE	SOURCE	IDENTIFIER
**Bacterial and virus strains**		

VSV-G pseudotyped ΔG-luciferase	Kerafast	Cat# EH1020-PM

**Biological samples**		

“JN.1 infx” sera	This paper and Wang et al.^14^	N/A
“KP.2 MV” sera	This paper and Wang et al.^15^	N/A

**Chemicals, peptides, and recombinant proteins**		

Polyethylenimine (PEI)	Polysciences Inc.	Cat# 23966-100
hACE2	This paper	N/A

**Critical commercial assays**		

Luciferase Assay System	Promega	Cat# E4550

**Experimental models: cell lines**		

HEK293T	ATCC	Cat# CRL-3216; RRID: CVCL_0063
Vero-E6	ATCC	Cat# CRL-1586; RRID: CVCL_0574

**Recombinant DNA**		

pCMV3-JN.1	Wang et al.^16^	N/A
pCMV3-KP.2	Wang et al.^14^	N/A
pCMV3-KP.3	Wang et al.^14^	N/A
pCMV3-KP.3.1.1	Wang et al.^13^	N/A
pCMV3-XEC	This paper	N/A
pCMV3-KP.3-T22N	This paper	N/A
pCMV3-KP.3-F59S	This paper	N/A

**Software and algorithms**		

GraphPad Prism V.10	GraphPad Software Inc	https://www.graphpad.com/scientific-software/prism/
Pymol V2.5.4	Schrödinger, Inc.	https://www.pymol.org/
Racmacs V1.1.4	Smith et al.^17^	https://acorg.github.io/Racmacs/

### Experimental model and subject details

#### Clinical cohorts

Serum samples were collected as part of the VIVA study at the University of Michigan^14^^,^^18^ and as part of the “COVID-19 Persistence and Immunology Cohort (C-PIC)” study at Columbia University. Specimens were obtained following participant informed consent and in adherence to the protocols approved by the Institutional Review Board of the University of Michigan Medical School (protocol HUM00232359) and Columbia University (protocol AAAS9722), respectively.

In this study, serum samples were collected from two cohorts: 1) individuals with a recent JN.1 sublineage infection (“JN.1 infx”); and 2) individuals who had been administered the updated KP.2 monovalent booster (“KP.2 MV”). The majority of the study subjects were female, representing 88.2%, with an average age of 55.3 years. Serum samples were collected, on average, 54.8 days post-JN.1 sublineage infection and 30.5 days post-KP.2 booster. Demographic details, vaccination status, and serum collection timelines are summarized for each cohort in Tables S1 and S2.

#### Cell lines

HEK293T (ATCC, CRL-3216) cells and Vero-E6 cells (ATCC, CRL-1586) were cultured in Dulbecco’s modified Eagle’s medium (DMEM) supplemented with 10% heat-inactivated fetal bovine serum and 1% penicillin-streptomycin. All cell lines were cultured in an atmosphere of 5% CO_2_ at 37°C.

### Method details

#### Construction of SARS-CoV-2 spike plasmids

The spike constructs of JN.1, KP.2, and KP.3 were generated as previously reported.^14^ The spike gene of KP.3.1.1 and XEC, as well as spike gene constructs bearing mutations T22N and F59S, were generated using Q5 site-directed mutagenesis with the KLD master mix kit (NEB). All constructs were confirmed by Sanger sequencing.

#### Pseudovirus production

Pseudotyped SARS-CoV-2 was produced following a previously established protocol.^19^ HEK293T cells were first transfected with spike-encoding plasmids using 1 mg/mL of PEI-MAX (Polysciences, Inc.) and cultured for 24 h. The transfected HEK293T cells were then infected with VSV-G pseudotyped ΔG-luciferase virus (Kerafast, EH1020-PM) at a multiplicity of infection (MOI) of approximately 3–5. Two hours later, the cells were washed three times with complete culture medium and cultured in fresh medium for another 24 h. The transfection supernatant was then harvested and clarified by centrifugation at 2000 rpm for 10 min. Each viral stock was subsequently incubated with 20% I1 hybridoma (ATCC, CRL-2700) supernatant for 1 h at room temperature to neutralize contaminating VSV-G particle before measuring titers and making aliquots for storage at −80°C until use.

#### Serum and monoclonal antibody neutralization, and ACE2 inhibition assays

Pseudoviruses were titrated to standardize the viral input prior to each neutralization or inhibition assay. For neutralization assays, serum samples were inactivated at 56°C for 30 min before use, and the inactivated sera were diluted by a factor of 100 followed by a series of 7 4-fold serial dilutions. For monoclonal antibody neutralization assays, each antibody was diluted from 10 μg/mL with a dilution factor of five across 7 serial dilutions. For ACE2 inhibition assays, as reported in our study,^20^ soluble chimeric human ACE2 (hACE2), which contains ACE2 residues 1–732 and is fused to human IgG1 Fc, was diluted from 3 μg/mL with a dilution factor of three across 7 serial dilutions. Following this, pseudoviruses were added and incubated at 37°C for 1 h. As a control, wells containing only the pseudovirus were also prepared on each test plate. Subsequently, Vero-E6 cells were seeded at 40,000 cells per well and were incubated overnight at 37°C on either neutralization or inhibition test plates. Afterward, cellular lysis was conducted, and the resultant luciferase activity was quantified employing the Luciferase Assay System (Promega) in tandem with Tecan Infinite 200 PRO using i-control software v.3.9.1.0, in accordance with the manufacturer’s instructions. The serum dilution that inhibits 50% of virus entry (ID_50_), or the half-maximal inhibitory concentration (IC_50_) by antibody and hACE2, was calculated using nonlinear five-parameter dose-response curve fitting using GraphPad Prism v.10.3.

#### Antigenic cartography

Antigenic cartography for the JN.1 subvariants was conducted through the integration of ID_50_ titers from individual sera, as previously described.^20^^,^^21^^,^^22^ The visual representations were generated utilizing the Racmacs package (version 1.1.4, accessible at https://acorg.github.io/Racmacs/) within the R computational environment, version 4.0.3. The algorithmic optimization process was executed over 2,000 iterations, with the 'minimum column basis' set to 'none'.

### Quantification and statistical analysis

ID_50_ values for serum neutralization and IC_50_ values for monoclonal antibody neutralization and hACE2 inhibition were obtained from a five-parameter dose-response curve using GraphPad Prism v.10.3. Statistical analyses were conducted by Wilcoxon matched-pairs signed-rank tests in the same software. Levels of statistical significance are annotated as: ns, not significant; ^∗^*p* < 0.05; ^∗∗^*p* < 0.01; ^∗∗∗^*p* < 0.001 and ^∗∗∗∗^*p* < 0.0001.

## Supplemental information

Document S1. Tables S1–S3

Document S2. Article plus supplemental information
