# Supplementary material for: Antibody evasiveness of SARS-CoV-2 subvariants KP.3.1.1 and XEC
Source: Cell Rep. 2025 Apr 8;44(4):115543. doi: 10.1016/j.celrep.2025.115543 (PMC12014523; doi:10.1016/j.celrep.2025.115543)
Supplement: Document S1. Tables S1–S3 [file mmc1.pdf]

Cell Reports, Volume *44*

Supplemental information

Antibody evasiveness of SARS-CoV-2 subvariants KP.3.1.1 and XEC

Qian Wang, Yicheng Guo, Ian A. Mellis, Madeline Wu, Hiroshi Mohri, Carmen Gherasim, Riccardo Valdez, Lawrence J. Purpura, Michael T. Yin, Aubree Gordon, and David D. Ho

Supplemental Information

Contents

Materials and Methods 2
Clinical cohorts 2
Cell lines 2
Construction of SARS-CoV-2 spike plasmids 2
Pseudovirus production 2
Serum and monoclonal antibody neutralization, and ACE2 inhibition assays 3
Antigenic cartography 3
Quantification and statistical analysis 4
Acknowledgements 4
Author contributions 4
Declaration of interests 4
Table S1: Spike mutations across examined SARS-CoV-2 variants. 5
Table S2: Summary of clinical cohorts. 6
Table S3: Participant demographic, vaccine, and infection details. 7
Supplementary references 8

Materials and Methods

Clinical cohorts

Serum samples were collected as part of the VIVA study at the University of Michigan[1,2] and as part of the "COVID-19 Persistence and Immunology Cohort (C-PIC)" study at Columbia University. Specimens were obtained following participant informed consent and in adherence to the protocols approved by the Institutional Review Board of the University of Michigan Medical School (protocol HUM00232359) and Columbia University (protocol AAAS9722), respectively.

In this study, serum samples were collected from two cohorts: 1) individuals with a recent JN.1 sublineage infection ("JN.1 infx"); and 2) individuals who had been administered the updated KP.2 monovalent booster ("KP.2 MV"). The majority of the study subjects were female, representing 88.2%, with an average age of 55.3 years. Serum samples were collected, on average, 54.8 days post-JN.1 sublineage infection and 30.5 days post-KP.2 booster. Demographic details, vaccination status, and serum collection timelines are summarized for each cohort in **Table S1 and Table S2**. In the JN.1 infx cohort, participants tested positive for SARS-CoV-2 by nucleic acid amplification testing. For participants JN.1-11 and -12 (in Columbia C-PIC cohort), due to recent international travel, their sequences were Sanger sequenced and assigned to the JN.1 sublineage. For the other participants in the JN.1 infx cohort (from University of Michigan), JN.1-13 through -32, JN.1 sublineage infection was inferred using epidemiological data on the circulating variants in Ann Arbor, Michigan, USA.

Cell lines

HEK293T (ATCC, CRL-3216) cells and Vero-E6 cells (ATCC, CRL-1586) were cultured in Dulbecco's modified Eagle's medium (DMEM) supplemented with 10% heat-inactivated fetal bovine serum and 1% penicillin-streptomycin. All cell lines were cultured in an atmosphere of 5% CO_2 at 37°C.

Construction of SARS-CoV-2 spike plasmids

The spike constructs of JN.1, KP.2, and KP.3 were generated as previously reported[1]. The spike gene of KP.3.1.1 and XEC, as well as spike gene constructs bearing mutations T22N and F59S, were generated using Q5 site-directed mutagenesis with the KLD master mix kit (NEB). All constructs were confirmed by Sanger sequencing.

Pseudovirus production

Pseudotyped SARS-CoV-2 was produced following a previously established protocol[3] . HEK293T cells were first transfected with spike-encoding plasmids using 1 mg/mL of PEI-MAX (Polysciences, Inc.) and cultured for 24 hours. The transfected HEK293T cells were then infected with VSV-G pseudotyped ΔG-luciferase virus (Kerafast, EH1020-PM) at a multiplicity of infection (MOI) of approximately 3 to 5. Two hours later, the cells were washed three times with

complete culture medium and cultured in fresh medium for another 24 hours. The transfection supernatant was then harvested and clarified by centrifugation at 2000 rpm for 10 minutes. Each viral stock was subsequently incubated with 20% I1 hybridoma (ATCC, CRL-2700) supernatant for 1 hour at room temperature to neutralize contaminating VSV-G particle before measuring titers and making aliquots for storage at -80°C until use.

Protein expression and purification

To express monoclonal antibodies or human ACE2, gWiz-antibody or pcDNA3-sACE2-WT(732)-IgG1 (Addgene plasmid #154104) plasmid were transfected into Expi293 cells using PEI at a ratio of 1:3, and the supernatants were collected after five days. Antibodies and human ACE2 (hACE2) fused to a Fc tag were purified with Protein A Sepharose (Cytiva) following the manufacturer's directions. Molecular weight and purity were confirmed by SDS-PAGE protein electrophoresis prior to use.

Serum and monoclonal antibody neutralization, and ACE2 inhibition assays

Pseudoviruses were titrated to standardize the viral input prior to each neutralization or inhibition assay. For neutralization assays, serum samples were inactivated at 56°C for 30 minutes before use, and the inactivated sera were diluted by a factor of 100 followed by a series of 7 four-fold serial dilutions. For monoclonal antibody neutralization assays, each antibody was diluted from 10 μg/mL with a dilution factor of five across 7 serial dilutions. For ACE2 inhibition assays, as reported in our study[4], soluble chimeric human ACE2 (hACE2), which contains ACE2 residues 1-732 and is fused to human IgG1 Fc, was diluted from 3 μg/mL with a dilution factor of three across 7 serial dilutions. Negative controls with only medium were included in parallel with each replicate for each sample. Following this, pseudoviruses were added and incubated at 37 °C for 1 hour. As a control, wells containing only the pseudovirus were also prepared on each test plate. Subsequently, Vero-E6 cells were seeded at 40,000 cells per well and were incubated overnight at 37°C on either neutralization or inhibition test plates. Afterwards, cellular lysis was conducted, and the resultant luciferase activity was quantified employing the Luciferase Assay System (Promega) in tandem with Tecan Infinite® 200 PRO using i-control™ software v.3.9.1.0, in accordance with the manufacturer's instructions. The serum dilution that inhibits 50% of virus entry (ID_{50}), or the half-maximal inhibitory concentration (IC_{50}) by antibody and hACE2, was calculated using nonlinear five-parameter dose-response curve fitting using GraphPad Prism v.10.3.

Antigenic cartography

Antigenic cartography for the JN.1 subvariants was conducted through the integration of ID_{50} titers from individual sera, as previously described[4-6]. The visual representations were generated utilizing the Racmacs package (version 1.1.4, accessible at https://acorg.github.io/Racmacs/) within the R computational environment, version 4.0.3. The algorithmic optimization process was executed over 2,000 iterations, with the 'minimum column basis' set to 'none'.

Quantification and statistical analysis

ID_{50} values for serum neutralization and IC_{50} values for monoclonal antibody neutralization and hACE2 inhibition were obtained from a five-parameter dose-response curve using GraphPad Prism v.10.3. Statistical analyses were conducted by Wilcoxon matched-pairs signed-rank tests in the same software. Levels of statistical significance are annotated as: ns, not significant; $*p < 0.05$; $**p < 0.01$; $***p < 0.001$ and $****p < 0.0001$.

Acknowledgements

This study was supported by funding from the NIH SARS-CoV-2 Assessment of Viral Evolution (SAVE) Program (subcontract no. 0258-A700-4609 under federal contract no. 75N93021C00014 to D.D.H. and (subcontract GR0010139-PO024016 under federal contract no. 75N93021C00016) to A.G. and the Gates Foundation (project INV019355) to D.D.H., internal startup funding UR014016 from Columbia University to Y.G. K23 AI171263 to L.J.P., K24 AI155230 to M.T.Y. We thank all who contributed their data to the Global Initiative on Sharing All Influenza Data (GISAID).

We express our gratitude to Jayesh Shah, Amanda Castillo, Meredith McNairy and Antonia Sturizo for conducting the C-PIC study (Columbia), and to Zijin Chu, Theresa Kowalski-Dobson, Anna Buswinka, Gabe Simjanovski, Joseph Wendzinski, Mayurika Patel, Kathleen Lindsey, and Dawson Davis of the VIVA study team for conducting the VIVA study.

Author contributions

The study was conceptualized by Q.W., Y.G., I.A.M., and D.D.H. Experiments were conducted and data analyzed by Q.W., Y.G., I.A.M., and M.W. Project management was handled by Q.W. Serum samples were collected by H.M., C.G., R.V., L.J.P., M.T.Y., A.G., and their colleagues. The results were analyzed, and the manuscript was written by Q.W., Y.G., M.W., I.A.M., and D.D.H. All contributing authors have reviewed and endorsed the manuscript.

Declaration of interests

D.D.H. co-founded TaiMed Biologics and RenBio, and he serves as a consultant for WuXi Biologics and Brii Biosciences and is a board director at Vicarious Surgical. A.G. served as a member of the scientific advisory board for Janssen Pharmaceuticals and has consulted and serves on a scientific advisory board for Sanofi Pasteur. The remaining authors declare no conflicts of interest.

Table S1: Spike mutations across examined SARS-CoV-2 variants.

NTD

Wuhan-Hu-1	V16ins	T19	R21	T22	L24	P25	P26	A27	S31	S50	F59	H69	V70	V127	G142	Y144	F157	R158	N211	L212	V213	L216	H245	A264
JN.1	MPLF	I	T		Δ	Δ	Δ	S		L		Δ	Δ	F	D	Δ	S	G	Δ	I	G	F	N	D
KP.2	MPLF	I	T		Δ	Δ	Δ	S		L		Δ	Δ	F	D	Δ	S	G	Δ	I	G	F	N	D
KP.3	MPLF	I	T		Δ	Δ	Δ	S		L		Δ	Δ	F	D	Δ	S	G	Δ	I	G	F	N	D
KP.3.1.1	MPLF	I	T		Δ	Δ	Δ	S	Δ	L		Δ	Δ	F	D	Δ	S	G	Δ	I	G	F	N	D
XEC	MPLF	I	T	N	Δ	Δ	Δ	S		L	S	Δ	Δ	F	D	Δ	S	G	Δ	I	G	F	N	D

RBD

Wuhan-Hu-1	I332	G339	R346	K356	S371	S373	S375	T376	R403	D405	R408	K417	N440	V445	G446	N450	L452	L455	F456	N460	S477	T478	N481	V483	E484	F486	Q493	Q498	N501	Y505
JN.1	V	H		T	F	P	F	A	K	N	S	N	K	H	S	D	W	S		K	N	K	K	Δ	K	P	Q	R	Y	H
KP.2	V	H	T	T	F	P	F	A	K	N	S	N	K	H	S	D	W	S	L	K	N	K	K	Δ	K	P	Q	R	Y	H
KP.3	V	H		T	F	P	F	A	K	N	S	N	K	H	S	D	W	S	L	K	N	K	K	Δ	K	P	E	R	Y	H
KP.3.1.1	V	H		T	F	P	F	A	K	N	S	N	K	H	S	D	W	S	L	K	N	K	K	Δ	K	P	E	R	Y	H
XEC	V	H		T	F	P	F	A	K	N	S	N	K	H	S	D	W	S	L	K	N	K	K	Δ	K	P	E	R	Y	H

SD1 / SD2 / S2

Wuhan-Hu-1	SD1 E554	SD1 A570	SD2 D614	SD2 P621	SD2 H655	SD2 N679	SD2 P681	S2 N764	S2 D796	S2 S939	S2 Q954	S2 N969	S2 V1104	S2 P1143
JN.1	K	V	G	S	Y	K	R	K	Y	F	H	K		L
KP.2	K	V	G	S	Y	K	R	K	Y	F	H	K	L	L
KP.3	K	V	G	S	Y	K	R	K	Y	F	H	K	L	L
KP.3.1.1	K	V	G	S	Y	K	R	K	Y	F	H	K	L	L
XEC	K	V	G	S	Y	K	R	K	Y	F	H	K	L	L

Table S2: Summary of clinical cohorts.

infx, infection; MV, monovalent vaccine; WT, wildtype; BV, bivalent vaccine.

		**All participants**		**JN.1 infx**		**KP.2 MV**	
		No. or Mean	% or (range)	No. or Mean	% or (range)	No. or Mean	% or (range)
**Total**		34		22		12	
**Female**		30	88.2%	19	86.4%	11	91.7%
**Male**		4	11.8%	3	13.6%	1	8.3%
**Age**		55.3	(24, 81)	55.9	(33, 78)	54.5	(24, 81)
**No. Vaccines**	All vaccines	5.7	(2, 9)	5.1	(2, 9)	6.8	(6, 9)
	WT	3.5	(2, 5)	3.4	(2, 4)	3.8	(3, 5)
	BA.5 BV	0.9	(0, 2)	0.9	(0, 2)	1.0	(0, 1)
	XBB.1.5	0.9	(0, 2)	0.85	(0, 2)	1.0	(0, 2)
	KP. 2 MV	0.4	(0,1)	0	(0,0)	1	(1,1)
**No. Infections**		1.4	(0, 3)	1.6	(1, 3)	0.8	(0, 2)
**Sera Days Post Infection**		155.4	(0, 835)	54.8	(32, 87)	598.4	(0, 835)
**Sera Days Post Vaccination**		230.0	(20, 1346)	350.6	(20, 1346)	30.5	(25, 36)

Table S3: Participant demographic, vaccine, and infection details.

Vaccine formulations are denoted as wildtype (WT), BA.5 Bivalent (BA.5), XBB.1.5 monovalent (XBB.1.5), and KP.2 monovalent (KP.2). Vaccine manufacturers are denoted as Pfizer (P), Moderna (M), Janssen (J), Other (O), and Unknown (U). Yr, years; Infx, infection; Vax, vaccination; DPI, days post infection; DPV, days post vaccination.

ID	Age (Yr)	Sex	Race	No. Infx	No. Vax	DPI	DPV	Vaccine History
**JN.1 infx**								
JN.1-11	71	M	Asian	2	5	40	315	WT-P/WT-P/WT-J/BA.5-P/XBB.1.5-P
JN.1-12	52	F	Asian	2	3	39	327	WT-P/WT-P/XBB.1.5-P
JN.1-13	66	F	White	2	6	59	151	WT-P/WT-P/WT-P/WT-P/BA.5-P/XBB.1.5-M
JN.1-14	35	F	White	2	5	32	155	WT-P/WT-P/WT-P/BA.5-P/XBB.1.5-P
JN.1-15	49	F	White	2	5	35	127	WT-P/WT-P/WT-M/BA.5-P/XBB.1.5-P
JN.1-16	38	F	White	2	5	39	162	WT-P/WT-P/WT-P/BA.5-P/XBB.1.5-M
JN.1-17	78	F	White	3	6	77	169	WT-P/WT-P/WT-P/WT-P/BA.5-P/XBB.1.5-P
JN.1-18	33	F	White	1	4	81	203	WT-P/WT-P/WT-P/XBB.1.5-P
JN.1-19	71	F	White	1	9	80	20	WT-M/WT-M/WT-M/WT-M/WT-M/BA.5-M
JN.1-20	67	F	Asian	2	7	54	25	WT-P/WT-P/WT-P/WT-P/BA.5-P/XBB.1.5-M
JN.1-21	62	F	White	2	6	55	117	WT-P/WT-P/WT-P/WT-M/BA.5-M/XBB.1.5-P
JN.1-22	48	F	White	1	3	55	847	WT-P/WT-P/WT-P
JN.1-23	69	F	White	2	3	55	847	WT-P/WT-P/WT-P
JN.1-24	41	F	Asian	2	5	87	179	WT-P/WT-P/WT-P/BA.5-M/XBB.1.5-M
JN.1-25	65	F	White	1	4	65	218	WT-J/WT-J/BA.5-P/XBB.1.5-P
JN.1-26	50	M	White	2	2	67	1346	WT-P/WT-P
JN.1-27	78	M	White	1	7	39	191	WT-P/WT-P/WT-P/WT-P/BA.5-P/BA.5-P
JN.1-28	57	F	White	1	5	61	608	WT-P/WT-P/WT-P/BA.5-M/BA.5-P
JN.1-29	59	F	White	2	6	36	188	WT-M/WT-M/WT-M/WT-M/BA.5-P/XBB.1.5-M
JN.1-30	64	F	White	1	6	40	253	WT-P/WT-P/WT-P/WT-P/BA.5-P/XBB.1.5-P
JN.1-31	42	F	White	1	5	70	192	WT-P/WT-P/WT-P/BA.5-M/XBB.1.5-O
JN.1-32	34	F	Other	1	3	39	1013	WT-P/WT-P/WT-P
**KP.2 MV**								
KP.2-V1	24	F	White	1	6	835	28	WT-P/WT-P/WT-P/BA.5-M/XBB.1.5-P/KP.2-P
KP.2-V2	25	F	White	0	6	-	32	WT-P/WT-P/WT-M/BA.5-M/XBB.1.5-M/KP.2-P
KP.2-V3	81	F	White	1	7	662	31	WT-P/WT-P/WT-P/WT-M/BA.5-P/XBB.1.5-M/KP.2-M
KP.2-V4	74	F	White	2	7	-	33	WT-P/WT-P/WT-P/WT-P/BA.5-P/XBB.1.5-M/KP.2-P
KP.2-V6	33	F	White	2	6	335	35	WT-P/WT-U/WT-P/BA.5-P/XBB.1.5-P/KP.2-P
KP.2-V7	75	F	Asian	0	6	-	36	WT-M/WT-M/WT-M/WT-M/WT-M/KP.2-M
KP.2-V8	59	F	White	0	7	-	35	WT-P/WT-P/WT-P/WT-P/BA.5-P/XBB.1.5-M/KP.2-P
KP.2-V9	68	F	White	0	9	-	33	WT-P/WT-P/WT-P/WT-P/WT-P/BA.5-P/XBB.1.5-P/XBB.1.5-P/KP.2-P
KP.2-V10	78	F	White	0	9	-	31	WT-P/WT-P/WT-P/WT-M/BA.5-M/BA.5-M/XBB.1.5-M/XBB.1.5-M/KP.2-M
KP.2-V11	35	M	Asian	1	4	-	25	WT-P/WT-P/WT-P/KP.2-M
KP.2-V12	25	F	Asian	0	6	-	25	WT-P/WT-P/WT-P/BA.5-M/XBB.1.5-M/KP.2-M
KP.2-V13	70	F	Asian	3	8	585	17	WT-P/WT-P/WT-P/WT-P/BA.5-P/BA.5-P

Supplementary references

1. Wang Q, Mellis IA, Ho J, et al. Recurrent SARS-CoV-2 spike mutations confer growth advantages to select JN.1 sublineages. *Emerg Microbes Infect* 2024; **13**(1): 2402880.
2. Simon V, Kota V, Bloomquist RF, et al. PARIS and SPARTA: Finding the Achilles' Heel of SARS-CoV-2. *mSphere* 2022; **7**(3): e0017922.
3. Liu L, Wang P, Nair MS, et al. Potent neutralizing antibodies against multiple epitopes on SARS-CoV-2 spike. *Nature* 2020; **584**(7821): 450-6.
4. Wang Q, Guo Y, Liu L, et al. Antigenicity and receptor affinity of SARS-CoV-2 BA.2.86 spike. *Nature* 2023; **624**(7992): 639-44.
5. Wang Q, Guo Y, Iketani S, et al. Antibody evasion by SARS-CoV-2 Omicron subvariants BA.2.12.1, BA.4 and BA.5. *Nature* 2022; **608**(7923): 603-8.
6. Wang Q, Iketani S, Li Z, et al. Alarming antibody evasion properties of rising SARS-CoV-2 BQ and XBB subvariants. *Cell* 2023; **186**(2): 279-86 e8.
