# Supplementary material for: Antibody evasiveness of SARS-CoV-2 subvariants KP.3.1.1 and XEC
Source: Cell Rep. 2025 Apr 8;44(4):115543. doi: 10.1016/j.celrep.2025.115543 (PMC12014523; doi:10.1016/j.celrep.2025.115543)
Supplement: Document S2. Article plus supplemental information [file mmc2.pdf]

Cell Reports

Report

Antibody evasiveness of SARS-CoV-2 subvariants KP.3.1.1 and XEC

Graphical abstract

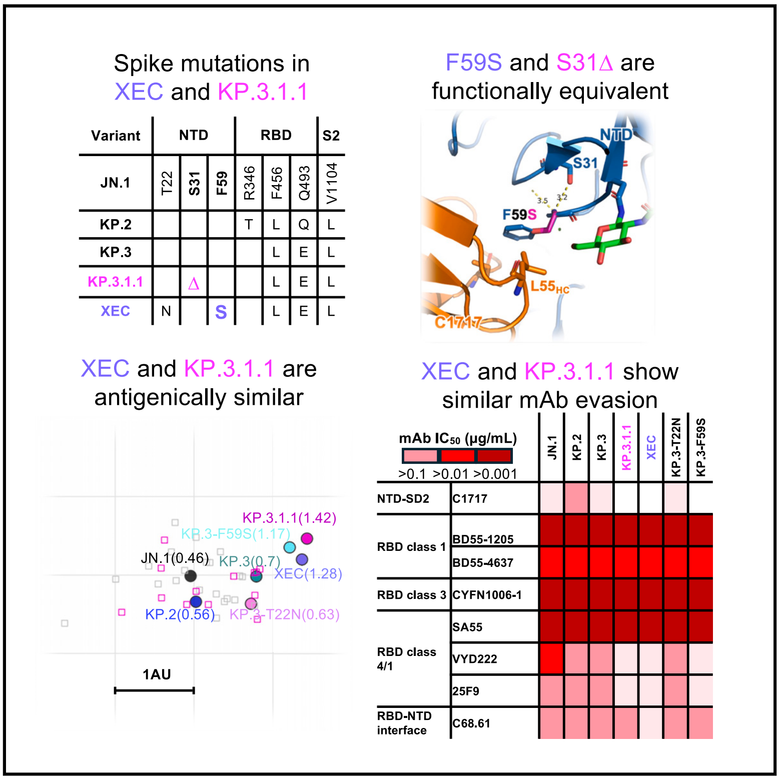

Highlights

- KP.3.1.1 and XEC are 1.3- to 1.6-fold more resistant to serum neutralization than KP.3
- KP.2 MV booster may elicit higher titers against KP.3.1.1 and XEC than JN.1 infection
- Spike mutations F59S and S31Δ are functionally similar for receptor binding and mAb evasion

Authors

Qian Wang, Yicheng Guo, Ian A. Mellis, ..., Michael T. Yin, Aubree Gordon, David D. Ho

Correspondence

dh2994@cumc.columbia.edu

In brief

SARS-CoV-2 Omicron JN.1 subvariants KP.3.1.1 and XEC emerged as dominant over parental strains. Wang et al. show that KP.3.1.1 and XEC are similarly antibody evasive due to different spike NTD mutations at interacting amino acids, which likely leads to similar impairments of spike conformational changes.

Wang et al., 2025, Cell Reports *44*, 115543
April 22, 2025 © 2025 The Author(s). Published by Elsevier Inc.
https://doi.org/10.1016/j.celrep.2025.115543

CellPress

Cell Reports

CellPress OPEN ACCESS

Report

Antibody evasiveness of SARS-CoV-2 subvariants KP.3.1.1 and XEC

Qian Wang,[1,2,8] Yicheng Guo,[1,8] Ian A. Mellis,[1,3,8] Madeline Wu,[1,8] Hiroshi Mohri,[1] Carmen Gherasim,[4] Riccardo Valdez,[4] Lawrence J. Purpura,[5] Michael T. Yin,[1,5] Aubree Gordon,[6] and David D. Ho[1,2,5,7,9,*]

[1]Aaron Diamond AIDS Research Center, Columbia University Vagelos College of Physicians and Surgeons, New York, NY, USA
[2]Pandemic Research Alliance Unit at the Wu Center for Pandemic Research, Columbia University Vagelos College of Physicians and Surgeons, New York, NY 10032, USA
[3]Department of Pathology and Cell Biology, Columbia University Vagelos College of Physicians and Surgeons, New York, NY 10032, USA
[4]Department of Pathology, University of Michigan, Ann Arbor, MI 48109, USA
[5]Division of Infectious Diseases, Department of Medicine, Columbia University Vagelos College of Physicians and Surgeons, New York, NY, USA
[6]Department of Epidemiology, University of Michigan, Ann Arbor, MI 48109, USA
[7]Department of Microbiology and Immunology, Columbia University Vagelos College of Physicians and Surgeons, New York, NY 10032, USA
[8]These authors contributed equally
[9]Lead contact
*Correspondence: dh2994@cumc.columbia.edu
https://doi.org/10.1016/j.celrep.2025.115543

SUMMARY

Severe acute respiratory syndrome coronavirus 2 (SARS-CoV-2) continues to evolve and spread, and it remains critical to understand the functional consequences of mutations in dominant viral variants. The recombinant JN.1 subvariant XEC recently replaced KP.3.1.1 to become the most prevalent subvariant worldwide. Here, we measure the *in vitro* neutralization of KP.3.1.1 and XEC by human sera, monoclonal antibodies, and the soluble human ACE2 (hACE2) receptor relative to the parental subvariants KP.3 and JN.1. KP.3.1.1 and XEC are slightly more resistant (1.3- to 1.6-fold) than KP.3 to serum neutralization and antigenically similar. Both also demonstrate greater resistance to neutralization by select monoclonal antibodies and soluble hACE2, all of which target the top of the viral spike. Our findings suggest that the upward motion of the receptor-binding domain in the spike may be partially hindered by the N-terminal domain mutations in KP.3.1.1 and XEC, allowing these subvariants to better evade serum antibodies that target the viral spike in the up position and to have a growth advantage.

INTRODUCTION

The severe acute respiratory syndrome coronavirus 2 (SARS-CoV-2) Omicron JN.1 subvariant rapidly increased in prevalence around the world starting in late 2023, and its progeny sublineages KP.2 and KP.3 were dominant successively and briefly thereafter. Recently, KP.3.1.1, bearing an S31 deletion (S31Δ) on top of the KP.3 spike, was dominant worldwide through October 2024[1] (Figure 1A). Since then, SARS-CoV-2 infections due to XEC, a recombinant of JN.1 subvariants KS.1 and KP.3.3, have become most frequent. XEC carries two additional spike N-terminal domain (NTD) mutations T22N and F59S beyond those in KP.3 (Figure 1B; Table S1). How these spike mutations confer a growth advantage to KP.3.1.1 and XEC remains unknown.

RESULTS

Serum neutralizing antibody evasion and antigenicity of KP.3.1.1 and XEC

To address this question, we first tested serum neutralization against vesicular stomatitis virus (VSV) pseudotyped KP.3.1.1, XEC, and XEC individual spike mutations on KP.3 (KP.3-T22N and KP.3-F59S) compared to JN.1, KP.2, and KP.3, using samples from two cohorts of adults: (1) participants with a history of JN.1 sublineage infection during 2024, sampled 32–87 days after infection ("JN.1 infx"), and (2) participants who received an updated KP.2-based mRNA monovalent vaccine booster, sampled approximately 4 weeks after dosing ("KP.2 MV") (Tables S2 and S3). The majority of participants were female. Compared with KP.3.1.1, XEC demonstrated a similar level of evasion to serum neutralization in the JN.1 infx cohort (Figure 1C) but was slightly more sensitive to serum neutralization in the KP.2 MV cohort (Figure 1D). KP.3 was 1.3- to 1.7-fold more resistant to serum neutralization than JN.1, while KP.3.1.1 and XEC were 1.3- to 1.6-fold more resistant than KP.3. Critically, we found that these increases in resistance to serum neutralization were explained by the component mutations S31Δ and F59S, individually tested on the background of KP.3 as KP.3.1.1 and KP.3-F59S, respectively (Figures 1C and 1D). In addition, serum neutralizing titers in KP.2 MV participants were generally higher than in JN.1 infx participants, with levels correlated with clinical protection.[2]

© 2025 The Author(s). Published by Elsevier Inc.
This is an open access article under the CC BY license (http://creativecommons.org/licenses/by/4.0/).

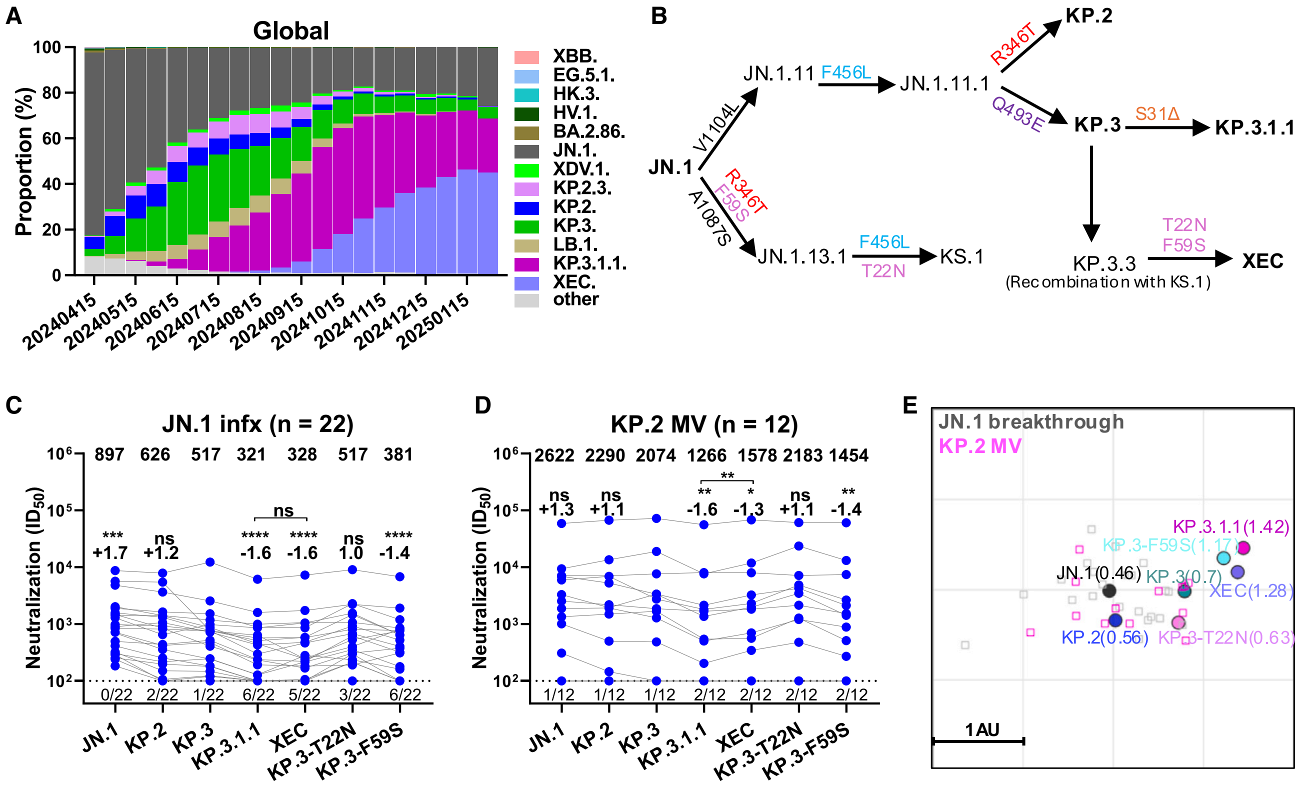

Figure 1. Characterization of SARS-CoV-2 JN.1 sublineages, including serum neutralizing antibody evasion and antigenicity

(A) Relative frequencies of dominant SARS-CoV-2 subvariants from April 2024 to February 2025; data are from GISAID.[1]

(B) Viral evolutionary pathways and spike mutations of the indicated JN.1 subvariants.

(C and D) Serum neutralizing titers (ID_{50}) against VSV-based pseudoviruses bearing spike proteins from SARS-CoV-2 JN.1 sublineages for samples from cohorts JN.1 infx (C) and KP.2 MV (D). Compared with KP.3, KP.3.1.1 carries an S31Δ spike mutation in the NTD. The geometric mean ID_{50} titer (GMT) is presented at the top. The fold change in GMT for each virus compared to KP.3 is also shown immediately above the symbols. Statistical analyses used Wilcoxon matched-pairs signed-rank tests, comparing to KP.3. *n*, sample size; ns, not significant. *$p < 0.05$, **$p < 0.01$, ***$p < 0.001$, ****$p < 0.0001$. Numbers under the dotted lines denote numbers of serum samples that were under the limit of detection ($ID_{50} < 100$).

(E) Antigenic map generated using all neutralization data from (C) and (D). One antigenic unit (AU) represents an approximately 2-fold change in ID_{50} titer. Serum samples and viruses are shown as squares and dots, respectively. The geometric mean antigenic distance of variants to sera is indicated in brackets.

To compare the antigenicity of the tested subvariants, the serum neutralization data from both cohorts were used to generate antigenic maps. We observed that KP.3.1.1, XEC, and KP.3-F59S clustered closely together, approximately 1.2 antigenic units from JN.1, while KP.3 and KP.3-T22N exhibited a shorter but similar antigenic distance to JN.1 (Figure 1E). These results indicate that F59S and S31Δ are comparable antigenically, while T22N has minimal impact on neutralization by these sera, consistent with other reports on the antigenicity of XEC and KP.3.1.1.[3,4]

Monoclonal antibody evasion and receptor binding of KP.3.1.1 and XEC

Next, to evaluate which epitopes were most closely associated with the antibody evasion of KP.3.1.1 and XEC, we performed neutralization assays using a panel of monoclonal antibodies (mAbs) that retained potency against KP.3, directed to multiple epitopes on the viral spike. S31Δ and F59S knocked out the NTD-SD2-specific antibody C1717[5] and impaired the receptor-binding domain (RBD) class 4/1 antibodies VYD222 (pemivibart)[6] and 25F9,[7] potentially explaining the increased resistance of KP.3.1.1 and XEC to serum neutralization (Figure 2A), consistent with other reports using distinct panels of mAbs.[3] But how do mutations at the bottom of the NTD affect mAbs directed to the top of the spike? To address this question, we tested the inhibition of soluble human ACE2 (hACE2) against the same panel of pseudoviruses. T22N did not alter the susceptibility of KP.3 to hACE2 inhibition, while S31Δ and F59S impaired hACE2 inhibition by 3.3- and 2.3-fold (Figure 2B), respectively, indicating a lower affinity for the viral receptor. These findings collectively suggested that the upward motion of the RBD may be impaired by either S31Δ or F59S, since the spike binding to both the receptor and class 4/1 antibodies requires the RBD to be in the up position. Indeed, structural analysis showed that S31 and F59 interact via hydrogen bonding in both the up and down conformations (Figure 2C) and that S31Δ and F59S mutations were functionally equivalent in altering NTD conformation, not only enabling escape from NTD-SD2-directed antibodies like C1717 but also likely indirectly hindering the upward movement of the RBD. S31Δ and

CellPress
OPEN ACCESS

A

IC_{50} (µg/mL)		JN.1	KP.2	KP.3	KP.3.1.1	XEC	KP.3-T22N	KP.3-F59S
NTD-SD2	C1717	1.485	0.669	1.084	>10	>10	1.563	>10
RBD class 1	BD55-1205	0.005	0.002	0.001	0.003	0.002	0.001	0.002
	BD55-4637	0.024	0.022	0.016	0.045	0.032	0.016	0.035
RBD class 3	CYFN1006-1	0.004	0.009	0.003	0.003	0.003	0.004	0.004
RBD class 4/1	SA55	0.003	0.003	0.002	0.003	0.003	0.002	0.003
	VYD222	0.096	0.121	0.415	2.675	1.753	0.512	2.009
	25F9	0.780	0.862	0.966	4.366	2.500	0.589	2.183
RBD-NTD interface	C68.61	0.677	0.456	0.537	0.998	1.025	0.320	0.776

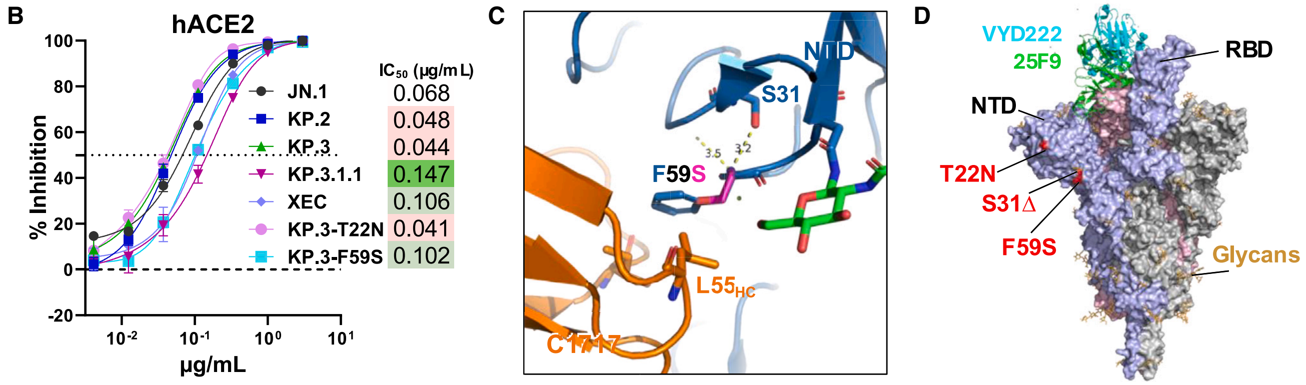

Figure 2. mAb neutralization, hACE2 inhibition, and structural analysis of the indicated JN.1 sublineage variants
(A) mAb neutralization against the indicated pseudoviruses. Antibody concentrations resulting in 50% inhibition of infectivity (IC_{50}) are presented.
(B) Sensitivity of JN.1 subvariants to hACE2 inhibition. IC_{50} values are also presented. Data are shown as mean ± SEM for three technical replicates.
(C) Structural analysis of S31Δ and F59S in the complex of C1717 and NTD (PDB: 7UAR).
(D) Structural analysis of T22N, S31Δ, and F59S in the SARS-CoV-2 spike (PDB: 7KRR), shown in complex with VYD222 (cyan; PDB: 7U2D) and 25F9 (green; PDB: 8GB5).

F59S, distant from the RBD, therefore likely indirectly impair binding of class 4/1 antibodies, in addition to their more direct interference with NTD-directed antibodies (Figure 2D). The conformational alterations caused by S31Δ and F59S may further enhance the immune evasion trend driven by F455S, F456L, and Q493E in the evolution of JN.1 sublineages (Figure 1B), which have already led to a large proportion of weak class 4/1 antibodies in the repertoire. This pattern recapitulates previous observations in XBB lineages, where a greater proportion of the RBD-down conformation in later subvariants contributes to immune evasion by conformational masking of immunodominant RBD regions.[8]

DISCUSSION

In summary, KP.3.1.1 and XEC demonstrate greater antibody evasion than JN.1 and KP.3, which likely contributes to their increasing global prevalence. The functional equivalence of S31Δ and F59S mutations renders KP.3.1.1 and XEC antigenically similar. The results here are consistent with most other posted reports using different clinical cohorts,[4,9–12] and they extend beyond posted or published data in several ways. Specifically, we show neutralizing titers in a cohort of KP.2 mRNA vaccine-boosted human participants, which have not been reported previously in the literature, to the best of our knowledge. Furthermore, our results based on neutralization with a large panel of mAbs, combined with hACE2 inhibition assays and structural modeling, provide evidence of up-conformation impairment as a common functional consequence of S31Δ and F59S mutations. Lastly, our results show that XEC is more resistant to neutralization by the US Food and Drug Administration-authorized VYD222 antibody, consistent with our prior results for KP.3.1.1,[13] so the clinical efficacy of VYD222 should be monitored closely as XEC increases in prevalence.

Limitations of the study

There are several factors that may limit the generalizability of our findings. First, there were relatively fewer participants in the KP.2 MV cohort compared to the JN.1 infx cohort, so we may not be able to detect smaller differences between these two groups. Second, we use human ACE2 pseudovirus inhibition to assess receptor binding, but we do not have results from orthogonal methods for accessing receptor binding. Third, we infer conformational change impairments due to F59S and S31Δ due to mAb neutralization results, receptor binding assay results, and computational structural analyses, but we do not present direct biochemical evidence of differences in the frequency of up vs. down spike conformational changes.

RESOURCE AVAILABILITY

Lead contact

Requests for further information, resources, and reagents should be directed to and will be fulfilled by the lead contact, David D. Ho (dh2994@cumc.columbia.edu).

Materials availability

All reagents generated in this study are available from the lead contact with a completed materials transfer agreement.

Data and code availability

- Data reported in this paper will be shared by the lead contact upon request.
- This paper does not report original code.
- Any additional information required to reanalyze the data reported in this paper is available from the lead contact upon request.

ACKNOWLEDGMENTS

This study was supported by funding from the NIH SARS-CoV-2 Assessment of Viral Evolution (SAVE) Program (subcontract 0258-A700-4609 under federal contract 75N93021C00014 to D.D.H. and subcontract GR0010139-PO024016 under federal contract 75N93021C00016 to A.G.) and the Gates Foundation (project INV019355 to D.D.H.), internal startup funding (UR014016) from Columbia University (to Y.G.), K23 AI171263 (to L.J.P.), and K24 AI155230 (to M.T.Y.). We thank all who contributed their data to the Global Initiative on Sharing All Influenza Data (GISAID).[1] We express our gratitude to Jayesh Shah, Amanda Castillo, Meredith McNairy, and Antonia Sturizo for conducting the C-PIC study (Columbia) and Zijin Chu, Theresa Kowalski-Dobson, Anna Buswinka, Gabe Simjanovski, Joseph Wendzinski, Mayurika Patel, Kathleen Lindsey, and Dawson Davis of the VIVA study team for conducting the VIVA study.

AUTHOR CONTRIBUTIONS

The study was conceptualized by Q.W., Y.G., I.A.M., and D.D.H. Experiments were conducted and data analyzed by Q.W., Y.G., I.A.M., and M.W. Project management was handled by Q.W. Serum samples were collected by H.M., C.G., R.V., L.J.P., M.T.Y., A.G., and their colleagues. The results were analyzed and the manuscript was written by Q.W., Y.G., M.W., I.A.M., and D.D.H. All contributing authors have reviewed and endorsed the manuscript.

DECLARATION OF INTERESTS

D.D.H. co-founded TaiMed Biologics and RenBio, and he serves as a consultant for WuXi Biologics and Brii Biosciences and is a board director at Vicarious Surgical. A.G. served as a member of the scientific advisory board for Janssen Pharmaceuticals and has consulted and serves on a scientific advisory board for Sanofi Pasteur.

STAR★METHODS

Detailed methods are provided in the online version of this paper and include the following:

- KEY RESOURCES TABLE
- EXPERIMENTAL MODEL AND SUBJECT DETAILS
  - Clinical cohorts
  - Cell lines
- METHOD DETAILS
  - Construction of SARS-CoV-2 spike plasmids
  - Pseudovirus production
  - Serum and monoclonal antibody neutralization, and ACE2 inhibition assays
  - Antigenic cartography
- QUANTIFICATION AND STATISTICAL ANALYSIS

SUPPLEMENTAL INFORMATION

Supplemental information can be found online at https://doi.org/10.1016/j.celrep.2025.115543.

Received: November 26, 2024
Revised: February 24, 2025
Accepted: March 19, 2025
Published: April 8, 2025

REFERENCES

1. Shu, Y., and McCauley, J. (2017). GISAID: Global initiative on sharing all influenza data - from vision to reality. Euro Surveill. *22*, 30494. https://doi.org/10.2807/1560-7917.ES.2017.22.13.30494.
2. Earle, K.A., Ambrosino, D.M., Fiore-Gartland, A., Goldblatt, D., Gilbert, P.B., Siber, G.R., Dull, P., and Plotkin, S.A. (2021). Evidence for antibody as a protective correlate for COVID-19 vaccines. Vaccine *39*, 4423–4428. https://doi.org/10.1016/j.vaccine.2021.05.063.
3. Liu, J., Yu, Y., Jian, F., Yang, S., Song, W., Wang, P., Yu, L., Shao, F., and Cao, Y. (2025). Enhanced immune evasion of SARS-CoV-2 variants KP.3.1.1 and XEC through N-terminal domain mutations. Lancet Infect. Dis. *25*, e6–e7. https://doi.org/10.1016/S1473-3099(24)00738-2.
4. Li, P., Faraone, J.N., Hsu, C.C., Chamblee, M., Liu, Y., Zheng, Y.M., Xu, Y., Carlin, C., Horowitz, J.C., Mallampalli, R.K., et al. (2024). Immune Evasion, Cell-Cell Fusion, and Spike Stability of the SARS-CoV-2 XEC Variant: Role of Glycosylation Mutations at the N-terminal Domain. Preprint at bioRxiv. https://doi.org/10.1101/2024.11.12.623078.
5. Wang, Z., Muecksch, F., Cho, A., Gaebler, C., Hoffmann, H.H., Ramos, V., Zong, S., Cipolla, M., Johnson, B., Schmidt, F., et al. (2022). Analysis of memory B cells identifies conserved neutralizing epitopes on the N-terminal domain of variant SARS-Cov-2 spike proteins. Immunity *55*, 998–1012.e8. https://doi.org/10.1016/j.immuni.2022.04.003.
6. FDA (2024). FDA Roundup: March 22, 2024. https://www.fda.gov/news-events/press-announcements/fda-roundup-march-22-2024.
7. Feng, Y., Yuan, M., Powers, J.M., Hu, M., Munt, J.E., Arunachalam, P.S., Leist, S.R., Bellusci, L., Kim, J., Sprouse, K.R., et al. (2023). Broadly neutralizing antibodies against sarbecoviruses generated by immunization of macaques with an AS03-adjuvanted COVID-19 vaccine. Sci. Transl. Med. *15*, eadg7404. https://doi.org/10.1126/scitranslmed.adg7404.
8. Zhang, Q.E., Lindenberger, J., Parsons, R.J., Thakur, B., Parks, R., Park, C.S., Huang, X., Sammour, S., Janowska, K., Spence, T.N., et al. (2024). SARS-CoV-2 Omicron XBB lineage spike structures, conformations, antigenicity, and receptor recognition. Mol. Cell *84*, 2747–2764.e7. https://doi.org/10.1016/j.molcel.2024.06.028.
9. Liu, J., Yu, Y., Jian, F., Yang, S., Song, W., Wang, P., Yu, L., Shao, F., and Cao, Y. (2024). Enhanced immune evasion of SARS-CoV-2 KP.3.1.1 and XEC through NTD glycosylation. Preprint at bioRxiv. https://doi.org/10.1101/2024.10.23.619754.
10. Sano, K., Miyakawa, K., Kato, H., Kimura, Y., Goto, A., Ryo, A., Watanabe, S., and Hasegawa, H. (2024). Neutralizing antibody evasion of SARS-CoV-2 JN.1 derivatives KP.3, KP.3.1.1, LB.1, and XEC. Preprint at bioRxiv. https://doi.org/10.1101/2024.11.04.621772.
11. Arora, P., Happle, C., Kempf, A., Nehlmeier, I., Stankov, M.V., Dopfer-Jablonka, A., Behrens, G.M.N., Pöhlmann, S., and Hoffmann, M. (2024). Impact of JN.1 booster vaccination on neutralisation of SARS-CoV-2 variants KP.3.1.1 and XEC. Lancet Infect. Dis. *24*, e732–e733. https://doi.org/10.1016/S1473-3099(24)00688-1.
12. Uriu, K., Kaku, Y., Uwamino, Y., Fujiwara, H., Saito, F., and Sato, K. (2024). Robust antiviral humoral immunity induced by JN.1 monovalent mRNA vaccines against a broad range of SARS-CoV-2 Omicron subvariants including JN.1, KP.3.1.1 and XEC. Preprint at bioRxiv. https://doi.org/10.1101/2024.11.20.624471.

CellPress
OPEN ACCESS

13. Wang, Q., Guo, Y., Ho, J., and Ho, D.D. (2024). Activity of Research-Grade Pemivibart against Recent SARS-CoV-2 JN.1 Sublineages. N. Engl. J. Med. *391*, 1863–1864. https://doi.org/10.1056/NEJMc2410203.

14. Wang, Q., Mellis, I.A., Ho, J., Bowen, A., Kowalski-Dobson, T., Valdez, R., Katsamba, P.S., Wu, M., Lee, C., Shapiro, L., et al. (2024). Recurrent SARS-CoV-2 spike mutations confer growth advantages to select JN.1 sublineages. Emerg. Microb. Infect. *13*, 2402880. https://doi.org/10.1080/22221751.2024.2402880.

15. Wang, Q., Mellis, I.A., Wu, M., Bowen, A., Gherasim, C., Valdez, R., Shah, J.G., Purpura, L.J., Yin, M.T., Gordon, A., et al. (2025). KP.2-based monovalent mRNA vaccines robustly boost antibody responses to SARS-CoV-2. Lancet Infect. Dis. *25*, e133–e134. https://doi.org/10.1016/S1473-3099(25)00058-1.

16. Wang, Q., Guo, Y., Bowen, A., Mellis, I.A., Valdez, R., Gherasim, C., Gordon, A., Liu, L., and Ho, D.D. (2024). XBB.1.5 monovalent mRNA vaccine booster elicits robust neutralizing antibodies against XBB subvariants and JN.1. Cell Host Microbe *32*, 315–321.e3. https://doi.org/10.1016/j.chom.2024.01.014.

17. Smith, D.J., Lapedes, A.S., de Jong, J.C., Bestebroer, T.M., Rimmelzwaan, G.F., Osterhaus, A.D.M.E., and Fouchier, R.A.M. (2004). Mapping the antigenic and genetic evolution of influenza virus. Science *305*, 371–376. https://doi.org/10.1126/science.1097211.

18. Simon, V., Kota, V., Bloomquist, R.F., Hanley, H.B., Forgacs, D., Pahwa, S., Pallikkuth, S., Miller, L.G., Schaenman, J., Yeaman, M.R., et al. (2022). PARIS and SPARTA: Finding the Achilles' Heel of SARS-CoV-2. mSphere *7*, e0017922. https://doi.org/10.1128/msphere.00179-22.

19. Liu, L., Wang, P., Nair, M.S., Yu, J., Rapp, M., Wang, Q., Luo, Y., Chan, J.F.W., Sahi, V., Figueroa, A., et al. (2020). Potent neutralizing antibodies against multiple epitopes on SARS-CoV-2 spike. Nature *584*, 450–456. https://doi.org/10.1038/s41586-020-2571-7.

20. Wang, Q., Guo, Y., Liu, L., Schwanz, L.T., Li, Z., Nair, M.S., Ho, J., Zhang, R.M., Iketani, S., Yu, J., et al. (2023). Antigenicity and receptor affinity of SARS-CoV-2 BA.2.86 spike. Nature *624*, 639–644. https://doi.org/10.1038/s41586-023-06750-w.

21. Wang, Q., Guo, Y., Iketani, S., Nair, M.S., Li, Z., Mohri, H., Wang, M., Yu, J., Bowen, A.D., Chang, J.Y., et al. (2022). Antibody evasion by SARS-CoV-2 Omicron subvariants BA.2.12.1, BA.4 and BA.5. Nature *608*, 603–608. https://doi.org/10.1038/s41586-022-05053-w.

22. Wang, Q., Iketani, S., Li, Z., Liu, L., Guo, Y., Huang, Y., Bowen, A.D., Liu, M., Wang, M., Yu, J., et al. (2023). Alarming antibody evasion properties of rising SARS-CoV-2 BQ and XBB subvariants. Cellule *186*, 279–286.e8. https://doi.org/10.1016/j.cell.2022.12.018.

STAR★METHODS

KEY RESOURCES TABLE

REAGENT or RESOURCE	SOURCE	IDENTIFIER
Bacterial and virus strains		
VSV-G pseudotyped ΔG-luciferase	Kerafast	Cat# EH1020-PM
Biological samples		
"JN.1 infx" sera	This paper and Wang et al.[14]	N/A
"KP.2 MV" sera	This paper and Wang et al.[15]	N/A
Chemicals, peptides, and recombinant proteins		
Polyethylenimine (PEI)	Polysciences Inc.	Cat# 23966-100
hACE2	This paper	N/A
Critical commercial assays		
Luciferase Assay System	Promega	Cat# E4550
Experimental models: cell lines		
HEK293T	ATCC	Cat# CRL-3216; RRID: CVCL_0063
Vero-E6	ATCC	Cat# CRL-1586; RRID: CVCL_0574
Recombinant DNA		
pCMV3-JN.1	Wang et al.[16]	N/A
pCMV3-KP.2	Wang et al.[14]	N/A
pCMV3-KP.3	Wang et al.[14]	N/A
pCMV3-KP.3.1.1	Wang et al.[13]	N/A
pCMV3-XEC	This paper	N/A
pCMV3-KP.3-T22N	This paper	N/A
pCMV3-KP.3-F59S	This paper	N/A
Software and algorithms		
GraphPad Prism V.10	GraphPad Software Inc	https://www.graphpad.com/scientific-software/prism/
Pymol V2.5.4	Schrödinger, Inc.	https://www.pymol.org/
Racmacs V1.1.4	Smith et al.[17]	https://acorg.github.io/Racmacs/

EXPERIMENTAL MODEL AND SUBJECT DETAILS

Clinical cohorts

Serum samples were collected as part of the VIVA study at the University of Michigan[14,18] and as part of the "COVID-19 Persistence and Immunology Cohort (C-PIC)" study at Columbia University. Specimens were obtained following participant informed consent and in adherence to the protocols approved by the Institutional Review Board of the University of Michigan Medical School (protocol HUM00232359) and Columbia University (protocol AAAS9722), respectively.

In this study, serum samples were collected from two cohorts: 1) individuals with a recent JN.1 sublineage infection ("JN.1 infx"); and 2) individuals who had been administered the updated KP.2 monovalent booster ("KP.2 MV"). The majority of the study subjects were female, representing 88.2%, with an average age of 55.3 years. Serum samples were collected, on average, 54.8 days post-JN.1 sublineage infection and 30.5 days post-KP.2 booster. Demographic details, vaccination status, and serum collection timelines are summarized for each cohort in Tables S1 and S2.

Cell lines

HEK293T (ATCC, CRL-3216) cells and Vero-E6 cells (ATCC, CRL-1586) were cultured in Dulbecco's modified Eagle's medium (DMEM) supplemented with 10% heat-inactivated fetal bovine serum and 1% penicillin-streptomycin. All cell lines were cultured in an atmosphere of 5% CO_2 at 37°C.

METHOD DETAILS

Construction of SARS-CoV-2 spike plasmids

The spike constructs of JN.1, KP.2, and KP.3 were generated as previously reported.[14] The spike gene of KP.3.1.1 and XEC, as well as spike gene constructs bearing mutations T22N and F59S, were generated using Q5 site-directed mutagenesis with the KLD master mix kit (NEB). All constructs were confirmed by Sanger sequencing.

Pseudovirus production

Pseudotyped SARS-CoV-2 was produced following a previously established protocol.[19] HEK293T cells were first transfected with spike-encoding plasmids using 1 mg/mL of PEI-MAX (Polysciences, Inc.) and cultured for 24 h. The transfected HEK293T cells were then infected with VSV-G pseudotyped ΔG-luciferase virus (Kerafast, EH1020-PM) at a multiplicity of infection (MOI) of approximately 3–5. Two hours later, the cells were washed three times with complete culture medium and cultured in fresh medium for another 24 h. The transfection supernatant was then harvested and clarified by centrifugation at 2000 rpm for 10 min. Each viral stock was subsequently incubated with 20% I1 hybridoma (ATCC, CRL-2700) supernatant for 1 h at room temperature to neutralize contaminating VSV-G particle before measuring titers and making aliquots for storage at −80°C until use.

Serum and monoclonal antibody neutralization, and ACE2 inhibition assays

Pseudoviruses were titrated to standardize the viral input prior to each neutralization or inhibition assay. For neutralization assays, serum samples were inactivated at 56°C for 30 min before use, and the inactivated sera were diluted by a factor of 100 followed by a series of 7 4-fold serial dilutions. For monoclonal antibody neutralization assays, each antibody was diluted from 10 μg/mL with a dilution factor of five across 7 serial dilutions. For ACE2 inhibition assays, as reported in our study,[20] soluble chimeric human ACE2 (hACE2), which contains ACE2 residues 1–732 and is fused to human IgG1 Fc, was diluted from 3 μg/mL with a dilution factor of three across 7 serial dilutions. Following this, pseudoviruses were added and incubated at 37°C for 1 h. As a control, wells containing only the pseudovirus were also prepared on each test plate. Subsequently, Vero-E6 cells were seeded at 40,000 cells per well and were incubated overnight at 37°C on either neutralization or inhibition test plates. Afterward, cellular lysis was conducted, and the resultant luciferase activity was quantified employing the Luciferase Assay System (Promega) in tandem with Tecan Infinite 200 PRO using i-control software v.3.9.1.0, in accordance with the manufacturer's instructions. The serum dilution that inhibits 50% of virus entry (ID_{50}), or the half-maximal inhibitory concentration (IC_{50}) by antibody and hACE2, was calculated using nonlinear five-parameter dose-response curve fitting using GraphPad Prism v.10.3.

Antigenic cartography

Antigenic cartography for the JN.1 subvariants was conducted through the integration of ID_{50} titers from individual sera, as previously described.[20–22] The visual representations were generated utilizing the Racmacs package (version 1.1.4, accessible at https://acorg.github.io/Racmacs/) within the R computational environment, version 4.0.3. The algorithmic optimization process was executed over 2,000 iterations, with the 'minimum column basis' set to 'none'.

QUANTIFICATION AND STATISTICAL ANALYSIS

ID_{50} values for serum neutralization and IC_{50} values for monoclonal antibody neutralization and hACE2 inhibition were obtained from a five-parameter dose-response curve using GraphPad Prism v.10.3. Statistical analyses were conducted by Wilcoxon matched-pairs signed-rank tests in the same software. Levels of statistical significance are annotated as: ns, not significant; *$p < 0.05$; **$p < 0.01$; ***$p < 0.001$ and ****$p < 0.0001$.

Cell Reports, Volume *44*

Supplemental information

Antibody evasiveness of SARS-CoV-2 subvariants KP.3.1.1 and XEC

Qian Wang, Yicheng Guo, Ian A. Mellis, Madeline Wu, Hiroshi Mohri, Carmen Gherasim, Riccardo Valdez, Lawrence J. Purpura, Michael T. Yin, Aubree Gordon, and David D. Ho

Supplemental Information

Contents

Materials and Methods 2
Clinical cohorts 2
Cell lines 2
Construction of SARS-CoV-2 spike plasmids 2
Pseudovirus production 2
Serum and monoclonal antibody neutralization, and ACE2 inhibition assays 3
Antigenic cartography 3
Quantification and statistical analysis 4
Acknowledgements 4
Author contributions 4
Declaration of interests 4
Table S1: Spike mutations across examined SARS-CoV-2 variants. 5
Table S2: Summary of clinical cohorts. 6
Table S3: Participant demographic, vaccine, and infection details. 7
Supplementary references 8

Materials and Methods

Clinical cohorts

Serum samples were collected as part of the VIVA study at the University of Michigan[1,2] and as part of the "COVID-19 Persistence and Immunology Cohort (C-PIC)" study at Columbia University. Specimens were obtained following participant informed consent and in adherence to the protocols approved by the Institutional Review Board of the University of Michigan Medical School (protocol HUM00232359) and Columbia University (protocol AAAS9722), respectively.

In this study, serum samples were collected from two cohorts: 1) individuals with a recent JN.1 sublineage infection ("JN.1 infx"); and 2) individuals who had been administered the updated KP.2 monovalent booster ("KP.2 MV"). The majority of the study subjects were female, representing 88.2%, with an average age of 55.3 years. Serum samples were collected, on average, 54.8 days post-JN.1 sublineage infection and 30.5 days post-KP.2 booster. Demographic details, vaccination status, and serum collection timelines are summarized for each cohort in **Table S1 and Table S2**. In the JN.1 infx cohort, participants tested positive for SARS-CoV-2 by nucleic acid amplification testing. For participants JN.1-11 and -12 (in Columbia C-PIC cohort), due to recent international travel, their sequences were Sanger sequenced and assigned to the JN.1 sublineage. For the other participants in the JN.1 infx cohort (from University of Michigan), JN.1-13 through -32, JN.1 sublineage infection was inferred using epidemiological data on the circulating variants in Ann Arbor, Michigan, USA.

Cell lines

HEK293T (ATCC, CRL-3216) cells and Vero-E6 cells (ATCC, CRL-1586) were cultured in Dulbecco's modified Eagle's medium (DMEM) supplemented with 10% heat-inactivated fetal bovine serum and 1% penicillin-streptomycin. All cell lines were cultured in an atmosphere of 5% CO_2 at 37°C.

Construction of SARS-CoV-2 spike plasmids

The spike constructs of JN.1, KP.2, and KP.3 were generated as previously reported[1]. The spike gene of KP.3.1.1 and XEC, as well as spike gene constructs bearing mutations T22N and F59S, were generated using Q5 site-directed mutagenesis with the KLD master mix kit (NEB). All constructs were confirmed by Sanger sequencing.

Pseudovirus production

Pseudotyped SARS-CoV-2 was produced following a previously established protocol[3] . HEK293T cells were first transfected with spike-encoding plasmids using 1 mg/mL of PEI-MAX (Polysciences, Inc.) and cultured for 24 hours. The transfected HEK293T cells were then infected with VSV-G pseudotyped ΔG-luciferase virus (Kerafast, EH1020-PM) at a multiplicity of infection (MOI) of approximately 3 to 5. Two hours later, the cells were washed three times with

complete culture medium and cultured in fresh medium for another 24 hours. The transfection supernatant was then harvested and clarified by centrifugation at 2000 rpm for 10 minutes. Each viral stock was subsequently incubated with 20% I1 hybridoma (ATCC, CRL-2700) supernatant for 1 hour at room temperature to neutralize contaminating VSV-G particle before measuring titers and making aliquots for storage at -80°C until use.

Protein expression and purification

To express monoclonal antibodies or human ACE2, gWiz-antibody or pcDNA3-sACE2-WT(732)-IgG1 (Addgene plasmid #154104) plasmid were transfected into Expi293 cells using PEI at a ratio of 1:3, and the supernatants were collected after five days. Antibodies and human ACE2 (hACE2) fused to a Fc tag were purified with Protein A Sepharose (Cytiva) following the manufacturer's directions. Molecular weight and purity were confirmed by SDS-PAGE protein electrophoresis prior to use.

Serum and monoclonal antibody neutralization, and ACE2 inhibition assays

Pseudoviruses were titrated to standardize the viral input prior to each neutralization or inhibition assay. For neutralization assays, serum samples were inactivated at 56°C for 30 minutes before use, and the inactivated sera were diluted by a factor of 100 followed by a series of 7 four-fold serial dilutions. For monoclonal antibody neutralization assays, each antibody was diluted from 10 μg/mL with a dilution factor of five across 7 serial dilutions. For ACE2 inhibition assays, as reported in our study[4], soluble chimeric human ACE2 (hACE2), which contains ACE2 residues 1-732 and is fused to human IgG1 Fc, was diluted from 3 μg/mL with a dilution factor of three across 7 serial dilutions. Negative controls with only medium were included in parallel with each replicate for each sample. Following this, pseudoviruses were added and incubated at 37 °C for 1 hour. As a control, wells containing only the pseudovirus were also prepared on each test plate. Subsequently, Vero-E6 cells were seeded at 40,000 cells per well and were incubated overnight at 37°C on either neutralization or inhibition test plates. Afterwards, cellular lysis was conducted, and the resultant luciferase activity was quantified employing the Luciferase Assay System (Promega) in tandem with Tecan Infinite® 200 PRO using i-control™ software v.3.9.1.0, in accordance with the manufacturer's instructions. The serum dilution that inhibits 50% of virus entry (ID_{50}), or the half-maximal inhibitory concentration (IC_{50}) by antibody and hACE2, was calculated using nonlinear five-parameter dose-response curve fitting using GraphPad Prism v.10.3.

Antigenic cartography

Antigenic cartography for the JN.1 subvariants was conducted through the integration of ID_{50} titers from individual sera, as previously described[4-6]. The visual representations were generated utilizing the Racmacs package (version 1.1.4, accessible at https://acorg.github.io/Racmacs/) within the R computational environment, version 4.0.3. The algorithmic optimization process was executed over 2,000 iterations, with the 'minimum column basis' set to 'none'.

Quantification and statistical analysis

ID_{50} values for serum neutralization and IC_{50} values for monoclonal antibody neutralization and hACE2 inhibition were obtained from a five-parameter dose-response curve using GraphPad Prism v.10.3. Statistical analyses were conducted by Wilcoxon matched-pairs signed-rank tests in the same software. Levels of statistical significance are annotated as: ns, not significant; $*p < 0.05$; $**p < 0.01$; $***p < 0.001$ and $****p < 0.0001$.

Acknowledgements

This study was supported by funding from the NIH SARS-CoV-2 Assessment of Viral Evolution (SAVE) Program (subcontract no. 0258-A700-4609 under federal contract no. 75N93021C00014 to D.D.H. and (subcontract GR0010139-PO024016 under federal contract no. 75N93021C00016) to A.G. and the Gates Foundation (project INV019355) to D.D.H., internal startup funding UR014016 from Columbia University to Y.G. K23 AI171263 to L.J.P., K24 AI155230 to M.T.Y. We thank all who contributed their data to the Global Initiative on Sharing All Influenza Data (GISAID).

We express our gratitude to Jayesh Shah, Amanda Castillo, Meredith McNairy and Antonia Sturizo for conducting the C-PIC study (Columbia), and to Zijin Chu, Theresa Kowalski-Dobson, Anna Buswinka, Gabe Simjanovski, Joseph Wendzinski, Mayurika Patel, Kathleen Lindsey, and Dawson Davis of the VIVA study team for conducting the VIVA study.

Author contributions

The study was conceptualized by Q.W., Y.G., I.A.M., and D.D.H. Experiments were conducted and data analyzed by Q.W., Y.G., I.A.M., and M.W. Project management was handled by Q.W. Serum samples were collected by H.M., C.G., R.V., L.J.P., M.T.Y., A.G., and their colleagues. The results were analyzed, and the manuscript was written by Q.W., Y.G., M.W., I.A.M., and D.D.H. All contributing authors have reviewed and endorsed the manuscript.

Declaration of interests

D.D.H. co-founded TaiMed Biologics and RenBio, and he serves as a consultant for WuXi Biologics and Brii Biosciences and is a board director at Vicarious Surgical. A.G. served as a member of the scientific advisory board for Janssen Pharmaceuticals and has consulted and serves on a scientific advisory board for Sanofi Pasteur. The remaining authors declare no conflicts of interest.

Table S1: Spike mutations across examined SARS-CoV-2 variants.

Wuhan-Hu-1	NTD																							
	V16ins	T19	R21	T22	L24	P25	P26	A27	S31	S50	F59	H69	V70	V127	G142	Y144	F157	R158	N211	L212	V213	L216	H245	A264
JN.1	MPLF	I	T		Δ	Δ	Δ	S		L		Δ	Δ	F	D	Δ	S	G	Δ	I	G	F	N	D
KP.2	MPLF	I	T		Δ	Δ	Δ	S		L		Δ	Δ	F	D	Δ	S	G	Δ	I	G	F	N	D
KP.3	MPLF	I	T		Δ	Δ	Δ	S		L		Δ	Δ	F	D	Δ	S	G	Δ	I	G	F	N	D
KP.3.1.1	MPLF	I	T		Δ	Δ	Δ	S	Δ	L		Δ	Δ	F	D	Δ	S	G	Δ	I	G	F	N	D
XEC	MPLF	I	T	N	Δ	Δ	Δ	S		L	S	Δ	Δ	F	D	Δ	S	G	Δ	I	G	F	N	D

Wuhan-Hu-1	RBD																													
	I332	G339	R346	K356	S371	S373	S375	T376	R403	D405	R408	K417	N440	V445	G446	N450	L452	L455	F456	N460	S477	T478	N481	V483	E484	F486	Q493	Q498	N501	Y505
JN.1	V	H		T	F	P	F	A	K	N	S	N	K	H	S	D	W	S		K	N	K	K	Δ	K	P	Q	R	Y	H
KP.2	V	H	T	T	F	P	F	A	K	N	S	N	K	H	S	D	W	S	L	K	N	K	K	Δ	K	P	Q	R	Y	H
KP.3	V	H		T	F	P	F	A	K	N	S	N	K	H	S	D	W	S	L	K	N	K	K	Δ	K	P	E	R	Y	H
KP.3.1.1	V	H		T	F	P	F	A	K	N	S	N	K	H	S	D	W	S	L	K	N	K	K	Δ	K	P	E	R	Y	H
XEC	V	H		T	F	P	F	A	K	N	S	N	K	H	S	D	W	S	L	K	N	K	K	Δ	K	P	E	R	Y	H

Wuhan-Hu-1	SD1		SD2					S2						
	E554	A570	D614	P621	H655	N679	P681	N764	D796	S939	Q954	N969	V1104	P1143
JN.1	K	V	G	S	Y	K	R	K	Y	F	H	K		L
KP.2	K	V	G	S	Y	K	R	K	Y	F	H	K	L	L
KP.3	K	V	G	S	Y	K	R	K	Y	F	H	K	L	L
KP.3.1.1	K	V	G	S	Y	K	R	K	Y	F	H	K	L	L
XEC	K	V	G	S	Y	K	R	K	Y	F	H	K	L	L

Table S2: Summary of clinical cohorts.

infx, infection; MV, monovalent vaccine; WT, wildtype; BV, bivalent vaccine.

		**All participants**		**JN.1 infx**		**KP.2 MV**	
		No. or Mean	% or (range)	No. or Mean	% or (range)	No. or Mean	% or (range)
**Total**		34		22		12	
**Female**		30	88.2%	19	86.4%	11	91.7%
**Male**		4	11.8%	3	13.6%	1	8.3%
**Age**		55.3	(24, 81)	55.9	(33, 78)	54.5	(24, 81)
**No. Vaccines**	All vaccines	5.7	(2, 9)	5.1	(2, 9)	6.8	(6, 9)
	WT	3.5	(2, 5)	3.4	(2, 4)	3.8	(3, 5)
	BA.5 BV	0.9	(0, 2)	0.9	(0, 2)	1.0	(0, 1)
	XBB.1.5	0.9	(0, 2)	0.85	(0, 2)	1.0	(0, 2)
	KP. 2 MV	0.4	(0,1)	0	(0,0)	1	(1,1)
**No. Infections**		1.4	(0, 3)	1.6	(1, 3)	0.8	(0, 2)
**Sera Days Post Infection**		155.4	(0, 835)	54.8	(32, 87)	598.4	(0, 835)
**Sera Days Post Vaccination**		230.0	(20, 1346)	350.6	(20, 1346)	30.5	(25, 36)

Table S3: Participant demographic, vaccine, and infection details.

Vaccine formulations are denoted as wildtype (WT), BA.5 Bivalent (BA.5), XBB.1.5 monovalent (XBB.1.5), and KP.2 monovalent (KP.2). Vaccine manufacturers are denoted as Pfizer (P), Moderna (M), Janssen (J), Other (O), and Unknown (U). Yr, years; Infx, infection; Vax, vaccination; DPI, days post infection; DPV, days post vaccination.

ID	Age (Yr)	Sex	Race	No. Infx	No. Vax	DPI	DPV	Vaccine History
**JN.1 infx**								
JN.1-11	71	M	Asian	2	5	40	315	WT-P/WT-P/WT-J/BA.5-P/XBB.1.5-P
JN.1-12	52	F	Asian	2	3	39	327	WT-P/WT-P/XBB.1.5-P
JN.1-13	66	F	White	2	6	59	151	WT-P/WT-P/WT-P/WT-P/BA.5-P/XBB.1.5-M
JN.1-14	35	F	White	2	5	32	155	WT-P/WT-P/WT-P/BA.5-P/XBB.1.5-P
JN.1-15	49	F	White	2	5	35	127	WT-P/WT-P/WT-M/BA.5-P/XBB.1.5-P
JN.1-16	38	F	White	2	5	39	162	WT-P/WT-P/WT-P/BA.5-P/XBB.1.5-M
JN.1-17	78	F	White	3	6	77	169	WT-P/WT-P/WT-P/WT-P/BA.5-P/XBB.1.5-P
JN.1-18	33	F	White	1	4	81	203	WT-P/WT-P/WT-P/XBB.1.5-P
JN.1-19	71	F	White	1	9	80	20	WT-M/WT-M/WT-M/WT-M/WT-M/BA.5-M
JN.1-20	67	F	Asian	2	7	54	25	WT-P/WT-P/WT-P/WT-P/BA.5-P/XBB.1.5-M
JN.1-21	62	F	White	2	6	55	117	WT-P/WT-P/WT-P/WT-M/BA.5-M/XBB.1.5-P
JN.1-22	48	F	White	1	3	55	847	WT-P/WT-P/WT-P
JN.1-23	69	F	White	2	3	55	847	WT-P/WT-P/WT-P
JN.1-24	41	F	Asian	2	5	87	179	WT-P/WT-P/WT-P/BA.5-M/XBB.1.5-M
JN.1-25	65	F	White	1	4	65	218	WT-J/WT-J/BA.5-P/XBB.1.5-P
JN.1-26	50	M	White	2	2	67	1346	WT-P/WT-P
JN.1-27	78	M	White	1	7	39	191	WT-P/WT-P/WT-P/WT-P/BA.5-P/BA.5-P
JN.1-28	57	F	White	1	5	61	608	WT-P/WT-P/WT-P/BA.5-M/BA.5-P
JN.1-29	59	F	White	2	6	36	188	WT-M/WT-M/WT-M/WT-M/BA.5-P/XBB.1.5-M
JN.1-30	64	F	White	1	6	40	253	WT-P/WT-P/WT-P/WT-P/BA.5-P/XBB.1.5-P
JN.1-31	42	F	White	1	5	70	192	WT-P/WT-P/WT-P/BA.5-M/XBB.1.5-O
JN.1-32	34	F	Other	1	3	39	1013	WT-P/WT-P/WT-P
**KP.2 MV**								
KP.2-V1	24	F	White	1	6	835	28	WT-P/WT-P/WT-P/BA.5-M/XBB.1.5-P/KP.2-P
KP.2-V2	25	F	White	0	6	-	32	WT-P/WT-P/WT-M/BA.5-M/XBB.1.5-M/KP.2-P
KP.2-V3	81	F	White	1	7	662	31	WT-P/WT-P/WT-P/WT-M/BA.5-P/XBB.1.5-M/KP.2-M
KP.2-V4	74	F	White	2	7	-	33	WT-P/WT-P/WT-P/WT-P/BA.5-P/XBB.1.5-M/KP.2-P
KP.2-V6	33	F	White	2	6	335	35	WT-P/WT-U/WT-P/BA.5-P/XBB.1.5-P/KP.2-P
KP.2-V7	75	F	Asian	0	6	-	36	WT-M/WT-M/WT-M/WT-M/WT-M/KP.2-M
KP.2-V8	59	F	White	0	7	-	35	WT-P/WT-P/WT-P/WT-P/BA.5-P/XBB.1.5-M/KP.2-P
KP.2-V9	68	F	White	0	9	-	33	WT-P/WT-P/WT-P/WT-P/WT-P/BA.5-P/XBB.1.5-P/XBB.1.5-P/KP.2-P
KP.2-V10	78	F	White	0	9	-	31	WT-P/WT-P/WT-P/WT-M/BA.5-M/BA.5-M/XBB.1.5-M/XBB.1.5-M/KP.2-M
KP.2-V11	35	M	Asian	1	4	-	25	WT-P/WT-P/WT-P/KP.2-M
KP.2-V12	25	F	Asian	0	6	-	25	WT-P/WT-P/WT-P/BA.5-M/XBB.1.5-M/KP.2-M
KP.2-V13	70	F	Asian	3	8	585	17	WT-P/WT-P/WT-P/WT-P/BA.5-P/BA.5-P

Supplementary references

1. Wang Q, Mellis IA, Ho J, et al. Recurrent SARS-CoV-2 spike mutations confer growth advantages to select JN.1 sublineages. *Emerg Microbes Infect* 2024; **13**(1): 2402880.
2. Simon V, Kota V, Bloomquist RF, et al. PARIS and SPARTA: Finding the Achilles' Heel of SARS-CoV-2. *mSphere* 2022; **7**(3): e0017922.
3. Liu L, Wang P, Nair MS, et al. Potent neutralizing antibodies against multiple epitopes on SARS-CoV-2 spike. *Nature* 2020; **584**(7821): 450-6.
4. Wang Q, Guo Y, Liu L, et al. Antigenicity and receptor affinity of SARS-CoV-2 BA.2.86 spike. *Nature* 2023; **624**(7992): 639-44.
5. Wang Q, Guo Y, Iketani S, et al. Antibody evasion by SARS-CoV-2 Omicron subvariants BA.2.12.1, BA.4 and BA.5. *Nature* 2022; **608**(7923): 603-8.
6. Wang Q, Iketani S, Li Z, et al. Alarming antibody evasion properties of rising SARS-CoV-2 BQ and XBB subvariants. *Cell* 2023; **186**(2): 279-86 e8.
